# Exploring meat processing in the past: Insights from the Nunamiut people

**DOI:** 10.1371/journal.pone.0245213

**Published:** 2021-01-13

**Authors:** Marie-Cécile Soulier

**Affiliations:** CNRS UMR 5608 TRACES, Université de Toulouse-Jean Jaurès, Maison de la Recherche, Toulouse, France; University at Buffalo - The State University of New York, UNITED STATES

## Abstract

Improving our knowledge of subsistence strategies and food processing techniques of past societies is of prime interest for better understanding human cultures as well as multiple aspects of human evolution. Beyond the simple matter of food itself, a substantial portion of socio-economic behavior is expressed in what, how, when, and with whom we eat. Over the last few decades, diverse methodologies for the analysis and interpretation of cut marks have progressively provided new insights for past butchery practices. For example, a recent study of the production of antelope biltong in South Africa concluded that the drying of meat generates high frequencies of longitudinal cut marks. This paper presents a cut mark analysis of faunal remains recovered by Lewis Binford from 8 campsites occupied by Nunamiut groups from the end of 19^th^ to middle of the 20^th^ century in the area around Anaktuvuk Pass, Alaska. The preparation of meat—primarily from caribou (*Rangifer tarandus*)–varied at these sites according to the season of occupation and was, depending on the site, either immediately consumed, processed after being stored in ice-cellars, or dried and stored. These faunal assemblages therefore provide a unique opportunity to explore the material traces of different meat preparation and preservation techniques in order to identify whether specific patterns can be identified and subsequently used to explore subsistence practices in the past. Binford’s Nunamiut faunal assemblages, which were produced by individuals using traditional techniques and methods, were analyzed in order to 1) further test the hypothesis that meat drying produces high frequencies of longitudinal cut marks, 2) explore the common assumption that skilled butchers leave smaller numbers of cut marks on bones compared to less experienced individuals, and 3) test whether cut mark patterns vary as a function of the processing techniques employed. The introduction of a %cutL index represents a quicker alternative to geo-referencing cut marks on bones when exploring meat processing techniques and methods and can easily be integrated in zooarchaeological analyses. While the results obtained support processing techniques linked to meat drying to leave high numbers of longitudinal cut marks, they are inconsistent with cut mark frequencies varying as a function of the butcher’s skill and experience. Analyzing cut mark patterns is therefore a reliable means for exploring food processing by past human societies and, by extension, their methods for safeguarding against unfavorable seasonal variations in both the abundance and condition of prey species. Identifying food storage in the archaeological record equally provides a unique window on to the social dynamics and potential inequalities of past societies.

## 1. Introduction

Better documenting subsistence strategies and associated techniques for the processing of vegetal and animal resources is essential for better understanding the specificities of human cultures. Food processing is also a key aspect for research on human evolution as it has been demonstrated that cooking food not only increases its overall energy content and digestibility while reducing bacterial growth but also broadens the spectrum of edible foods [1–6]. The preservation and storage of food is also of central interest amongst numerous food related issues for past societies. Although food storage has long been perceived as exclusive to agro-pastoral and sedentary societies, this practice has also been documented for nomadic hunter-gatherer groups [7–13]. Food-storage for these groups is intimately tied to questions surrounding subsistence planning, mobility patterns, and the anticipation of needs, and is therefore a fundamental aspect of the hunter-gatherer logistical organization. For societies that rely on the hunting of migratory species, storing surplus food helps secure against lean periods when prey is either fat-depleted and/or absent from the immediate environment. This is the case for present day populations inhabiting (sub-)arctic contexts where plant consumption is limited by a short growing season and the diet is almost entirely based on the intake of animal protein. The food storage practices of nomadic hunter-gatherers also have implications for group mobility patterns. Group movements are not only correlated with the quantity of products stored but also with the storage technique used. For example, dried meat is easily transported, as drying significantly reduces its weight and size [14], whereas the use of ice-cellars or other types of frozen storage, such as lakes, means returning to the storage site and therefore represents a constraint in the mobility system. In addition to these logistical aspects, storage also plays an important social function. First, storing food necessarily implies the acquisition of a mass of food that exceeds the immediate needs of the group. As food must be processed quickly so that it doesn’t spoil, the proper preparation of food stocks inevitably requires the cooperation of several individuals. Moreover, some storage processing techniques require special know-how and several forms of storage may have contributed to the emergence of specialists (or at least specializations) and a division of labor [15]. Finally, intra-group disparities in food storage may underlie the emergence of social inequalities [for a discussion see 8, 10] or, at the very least, are intimately connected to the appearance of complex social relations.

On multiple occasions, past hunter-gatherers confronted contrasting environmental conditions similar to those in which present-day groups regularly store food to avoid shortages in winter. Caches or structures that potentially served to store food are however rarely found in the archaeological record. This is primarily due to that fact that, ethnographically, these structures are commonly made from perishable materials or are unidentifiable, empty features. Moreover, food caches are frequently not on campsites but scattered across the landscape, and therefore unlikely to be located by archaeologists [9, 12, 13, 16–20]. Documenting meat-processing techniques in the past is also complicated by the fact that the meat itself does not preserve, meaning that archaeologists often have to rely on indirect evidence.

Cut marks on mammal bones were quickly identified as a clear indication of carcass processing [21, 22] and have thus been the subject of extensive study over the last few decades. Detailed taphonomic approaches built from experimental data and ethnographic observations allow genuine anthropogenic cut marks to be reliably distinguished from those generated by non-human agents [e.g. 23–32]. The micro-morphological analysis of cut marks and considerations of their frequency as well as the skeletal part concerned equally provide insights as to the type of tool used in butchery [31, 33–45] and allow archaeologists to explore links between cut marks and carcass size and freshness [35, 45–49]. These approaches also allow assessments of whether cut mark frequency reflects the butcher’s experience rather than the number of individuals involved [40, 49–51] or if it is influenced by bone fragmentation [52]. On the other hand, several studies have relied on the location of cut marks to infer butchery activities [53–61], and sound criteria are now available for identifying skinning, disarticulation, defleshing, and the extraction of tendons from both ungulates and small game. It has also been suggested that meat preparation techniques can influence cut mark orientation and frequency; for example, removing the easily-detachable cooked meat from the bone produces few cut marks [57] while, in South Africa, preparing meat for drying generates longitudinal cut marks more frequently compared to simple defleshing [62].

In order to better understand butchering practices and food consumption patterns of past societies, the present study focuses on faunal material recovered by Lewis Binford during his work with the semi-nomadic Nunamiut people of northern Alaska [9] who use traditional techniques and methods to hunt and butcher prey. The faunal assemblages result either from immediately consumed meat, meat prepared for drying, or meat stored in ice-cellars depending on the season of occupation. The frequency, orientation, and location of cut marks were recorded in order to 1) test whether the percentage of longitudinal cut marks is a reliable criterion for identifying meat drying in contexts other than South Africa and uniquely involving antelopes, and 2) to document patterns of cut marks resulting from other types of meat preparation (i.e. defleshing for immediate consumption and meat processed after frozen storage) that could be used to identify meat processing practices in the past, particularly for populations focused on the exploitation of wild reindeer/caribou, as is the case for many prehistoric societies. The faunal assemblages studied here result from a small group sharing similar technical know-how and includes two sites where a single individual prepared meat in two different ways. This dataset therefore makes it possible to test whether the observed differences reflect preparation methods rather than particular traditions or inter-individual variation.

## 2. Material and methods

**Fig 1:**
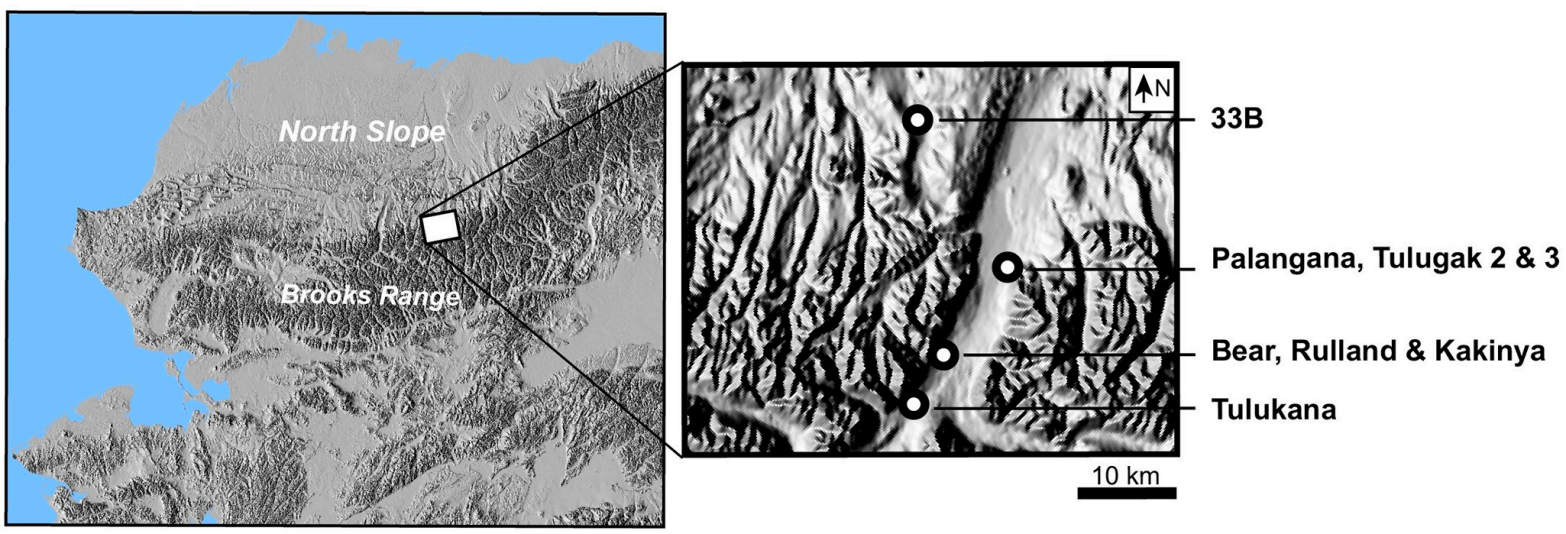
Location of the studied sites. Maps: National Atlas of the United States. (2005). Grayscale Alaska Shaded Relief—200-Meter Resolution. National Atlas of the United States. Available at: http://purl.stanford.edu/sb465jq2544. Public domain.

The faunal remains recovered by L. Binford during his work with Nunamiut in the early 1970s comprises primarily caribou remains, a species common on numerous archaeological sites. Cut mark frequencies and orientation were recorded on the meaty long bones of this species to determine whether meat-drying, the preparation of stored frozen meat, and the immediate consumption of freshly slaughtered animals leave distinctive cut mark patterns. All material was analyzed at the University of Arizona, where the faunal collection is housed. Eight sites from Binford’s work with the Nunamiut (*Kakinya*, *Rulland*, *Bear*, *Tulukana*, *Tulugak 3 & 2*, *33B* and a sample from *Palangana*) were studied, all of which are located in the Anaktuvuk Pass area of northern Alaska (Fig 1). *Kakinya*, *Rulland*, *Bear*, *33B*, and the two *Tulugak* sites were abandoned about 20 years before Binford’s began his work with the Nunamiut. He did, however, have the opportunity to interview the heads of families (Elijah Kakinya, Frank Rulland, and Simon Paneak) who occupied these sites and discuss the activities carried out at each. Unless otherwise stated, the contextual information related here can be found in Binford’s 1978 book *Nunamiut Ethnoarchaeology* [9]). Additional supporting information and/or reports concerning the daily life of this small Nunamiut group is available in several other publications [63–69].

Elijah Kakinya’s family and their 15 dogs occupied the site of *Kakinya* for a total of 39 days during two consecutive autumns in 1948 and 1949. *Rulland* was occupied at exactly the same time by 8 members of the Rulland family and their 14 dogs for 26 days. Both camps were used for hunting caribou (*Rangifer tarandus*) and, to a lesser extent, Dall sheep (*Ovis dalli*), as they were located on an autumn caribou migration route. At both camps, stocks of dry meat were built for the winter. The *Bear* site was occupied by the Kakinya family immediately after the site of *Kakinya*. At this winter camp the family primarily consumed meat that had previously been stored in ice-cellars as well as meat prepared at the previous campsite. The two *Tulugak* sites (*TL2* & *TL3*) were summer residential camps occupied between July and the end of September in 1948–49. Freshly slaughtered animals were primarily consumed alongside a smaller amount of dried meat at both sites. Site *33B* was a late summer hunting camp where slaughtered animals were immediately consumed by a single family over a period of one week in early September 1955. Portions of carcasses were fed to the families’ dogs at all of these sites.

*Tulukana* and *Palangana* were occupied around 1880, and no direct informants were alive when Binford conducted his interviews. However, informants led Binford to the settlements and provided detailed information about the season of occupation and the function of the sites. *Tulukana* is located a few miles north of Anaktuvuk Pass and was occupied during the fall. Here, caribou were driven towards Summit Lake and speared from a kayak, with the resulting meat subsequently dried. *Palangana* was a large winter encampment, east of Tulugak Lake, that comprised several houses.

**Fig 2:**
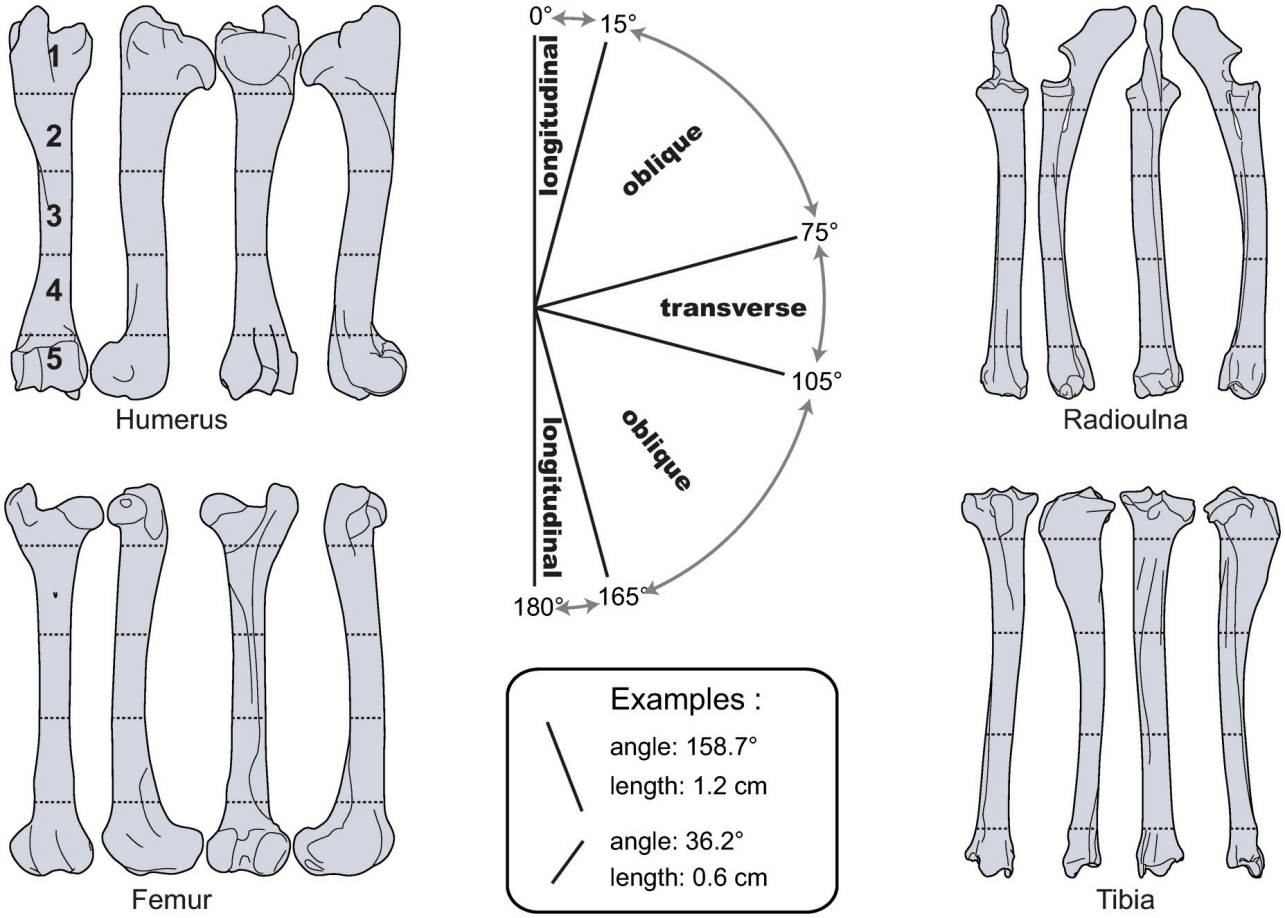
Bone templates showing long bone orientation, length, the 5 bone portions, and classes of cut mark orientation used in this study, from Soulier and Morin 2016 [62].

The NISP (Number of Identified Specimen), MNI (Minimum Number of Individuals) and MNE (Minimum Number of Elements) were calculated for all sites. For the site of Palangana, the MNI was calculated from teeth in mandibles recovered from the site, while the cut mark analysis focused uniquely on material from House 1, which was occupied by the same family for at least two successive winters. All identifiable remains were observed under low-angled light with a ×30 loupe. Taphonomic alterations, such as root etching, cracks, desquamation, dissolution, sheeting, and carnivore damages [e.g. 28, 70–72], were recorded for taxonomically identifiable remains. Only classic V-shaped cut marks located on caribou bones were considered in this study. Cut marks on the meat-bearing long bones of caribou (i.e., humerus, radioulna, femur, and tibia: Fig 2) were graphically recorded on bone templates with Adobe Illustrator^™^ using the methodology described by Soulier and Morin [62]. Cut mark position and orientation were recorded on a complete bone template for each element that was arbitrarily scaled to 19.84 cm. The QGIS software package (3.6 Noosa) was used to calculate cut mark length (expressed in log10) and orientation. The CRS EPSG:32662 –WGS 84 / Plate Carree was used in order to best limit terrestrial reprojection deformations. Cut marks were partitioned into 10 classes of log10-transformed lengths and 12 classes of orientation. Cut marks were considered longitudinal when oriented between 0–15° and 165–180°, oblique between 15–75° and 105–165°, and transverse for those oriented between 75° and 105°. Raw data are available in the (S1 Appendix). Isotropy in cut mark orientations for the different 15°classes were explored via rose diagrams created with Stereonet. Cut marks were assigned to one of 5 bone portions (Fig 2) according to their central point (calculated using the QGIS centroïd formula). If a cut mark intersected several bone portions it was assigned to the one containing its midpoint. The percentage of bone fragments from portions 2 to 4 bearing at least one longitudinal mark was calculated. This ratio (%cutL) was first calculated by adding all the fragments of the humerus, radioulna, femur, and tibia, and then for each of these bones individually. This calculation is, for example, *the number of femur fragments from portions 2 to 4 with longitudinal cut marks×100 / number of femur fragments from portions 2 to 4 with cut marks*. The ratio is based on the number of fragments with cut marks in order to generate data that are easily comparable between assemblages, sites, and observers. Bones without observable surfaces were excluded from the cut mark calculations. Finally, the reported seasons of occupation were compared with tooth wear and eruption sequences using data available in Miller [73], with the birthing season between the end of May to mid-June, as this is the case for the Porcupine and Central Arctic Caribou herds of the Brooks Range Mountains [74, 75]. Age estimation based on the degree of epiphyseal fusion were determined with reference to the remains of 4 month-old and a newborn caribou from the Anaktuvuk Pass area housed at the University of Arizona. Note that, for winter sites, seasonality data reflects the period of slaughter and not when the stored meat was consumed. Mortality profiles are presented using a caribou-adapted ternary diagram available in Discamps and Costamagno [76] with confidence ellipses calculated using the script provided by Steele and Weaver [77].

## 3. Results

### 3.1 General assemblage characteristics

Although the number of remains collected from each site is highly variable, caribou (*Rangifer tarandus*) is by far the dominant species in all assemblages (Table 1), and is accompanied by significantly smaller numbers of Dall sheep, elk, bear, fox, lagopedes, and rodent remains. The number of individuals varies substantially between sites, with high MNIs at *Palangana*, *Tulukana*, *Bear* and *Kakinya*, and much smaller numbers at *Rulland*, *33B*, *TL2* and *TL3* (Table 1).

**Table 1:** Faunal spectra for *Palangana*, *Bear*, *33B*, the two Tulugak sites (*TL2* & *TL3*), *Kakinya*, *Rulland* and *Tulukana* expressed in NISP.

	Palangana	Bear	33B	TL 2	TL 3	Kakinya	Rulland	Tulukana
*Rangifer tarandus*	277 (99/9)*	1637 (6/21)	136 (2/6)	140 (4/3)	882 (5/9)	1788 (13/18)	190 (3/7)	2536 (32/35)
*Ovis dalli*	X				1	215 (3/8)	7 (0/1)	11 (1/2)
*Alces alces*			4 (0/2)	4				
*Ursus* sp.		2 (0/1)						
*Vulpinae*					3			
*Canidae*	X							
*Rodentia*					1	1		
*Lagopus lagopus*	X							
*Galloanserae*					6			

The MNI based on dental and bone remains, respectively, are presented in parentheses.

*Note that for Palangana the studied material comprises meaty long bones from House 1 and all the mandibles recovered from the site, which explains the significant difference between the MNI calculated from teeth and bone.

**Fig 3:**
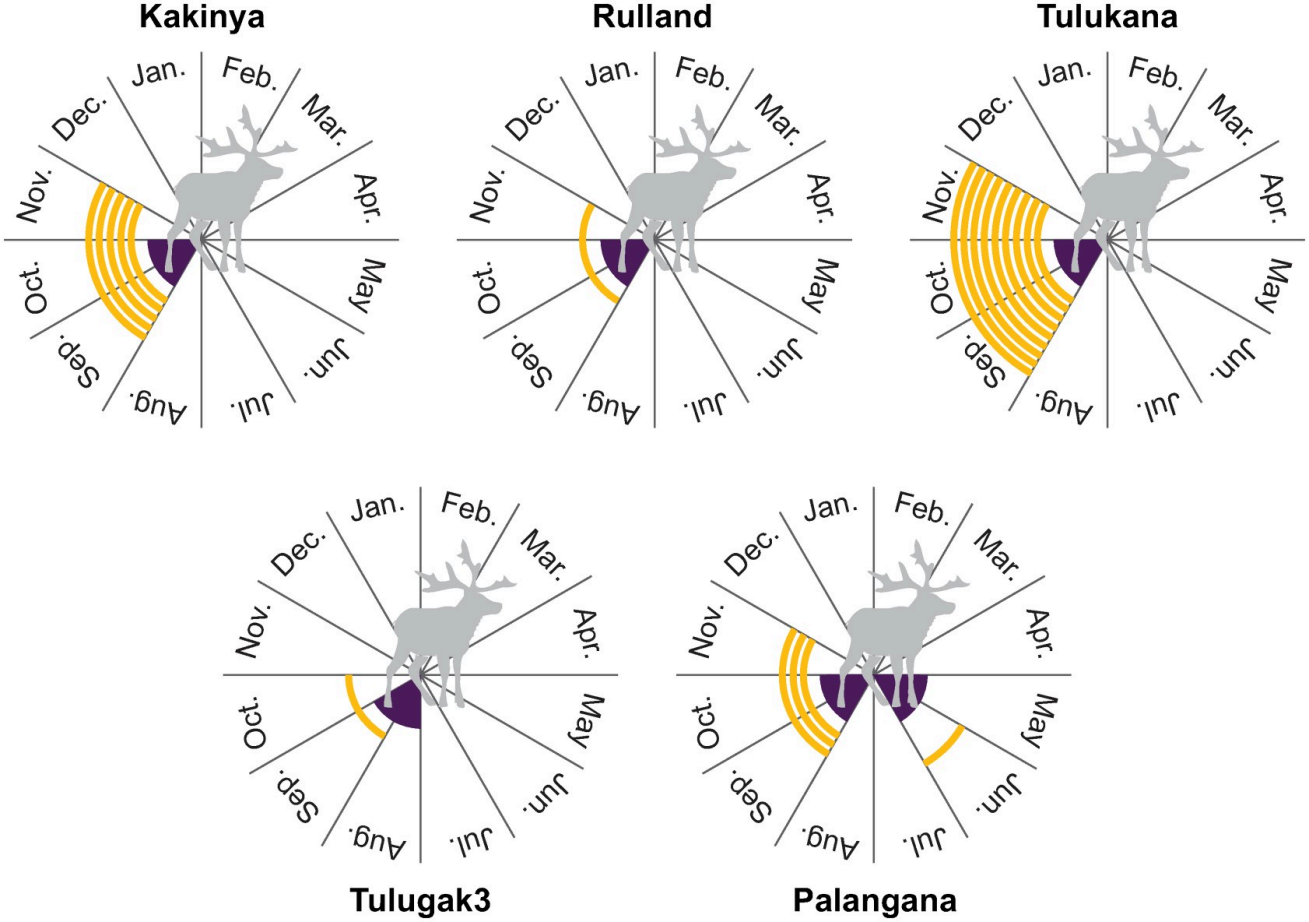
Seasonality data for *Kakinya*, *Rulland*, *Tulukana*, *TL3*, and *Palangana* (orange lines), and expected season (in the center of the circle, in purple) of caribou slaughtering according to Binford’s informants.

Seasonality data (Fig 3) from deciduous caribou teeth recovered from *Kakinya*, *Rulland*, and *Tulukana* confirm Binford’s informants’ accounts that these campsites were occupied during the autumn. The most precise seasonality evidence for these sites correspond to four different individuals slaughtered at the age of 3–5 months and one 15–17 months at *Kakinya*, one 3–5 months at *Rulland*, seven 3–5 months plus two 15–17 months at *Tulukana*. While determining the season of death for the *Tulugak 3* caribou is impossible due to the lack of dental remains, the size and epiphyseal stages (cf. *supra*) indicate at least one individual to have been 3–4 months old when slaughtered. The absence of dental evidence or remains of juvenile caribou precludes establishing the season during which the caribou at *Tulugak 2* and site *33B* were slaughtered. As noted above, seasonality data for winter campsites reveal when stocks were built, not when the meat was consumed. Newborn caribou remains (mandible with unworn milk teeth) and three individuals aged around 3 to 5 months from *Palangana* demonstrate, respectively, stores to have been established on sites occupied in the early summer and during the autumn migration. Data from the *Bear* site are too imprecise to determine if stocks were built-up only during the fall, as reported by Binford’s informants (estimated age-at-death ranges from 15 to 22 months).

**Fig 4:**
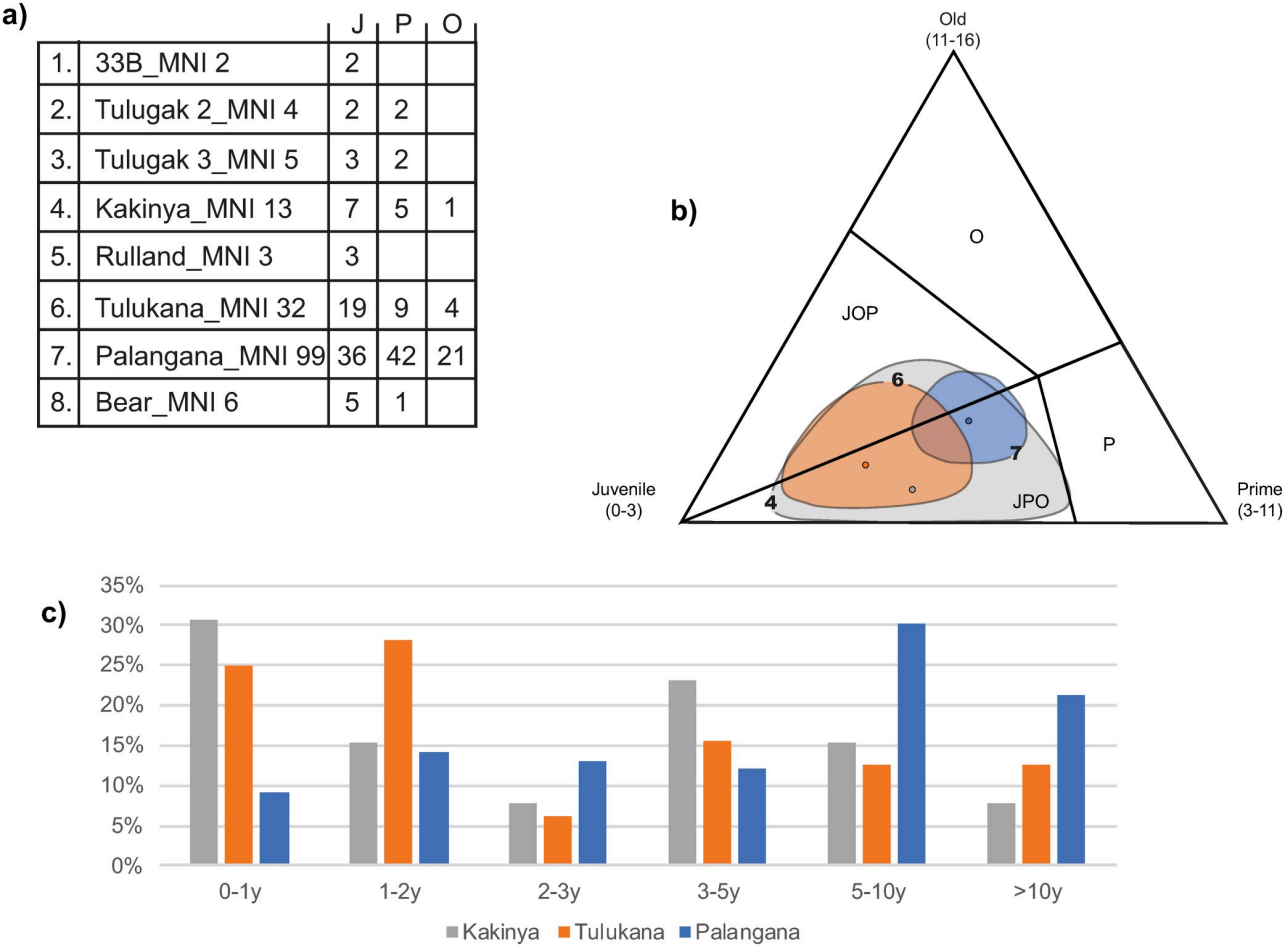
a) Mortality profiles for *Palangana*, *Bear*, *33B*, the two Tulugak sites (*TL2* & *TL3*), *Kakinya*, *Rulland*, and *Tulukana* by large ‘Juvenile, Prime, Old’ age-classes, b) ternary diagram for the three assemblages with large sample sizes (MNItot > 10) and c) subsequent detailed mortality profiles.

With the exception of *Palangana*, where adults are most frequent, young individuals are best represented at all of these campsites (Fig 4). However, when confidence ellipses are considered, no site falls within a specific zone of the ternary diagram, limiting our ability to explore hunting strategies.

Faunal remains are generally well preserved (Table 2), with only a few heavily-altered pieces classified as ‘unobservable’. *Tulukana* produced the most ‘unobservable’ remains due to the high proportion of bones with cortical surfaces bearing evidence for desquamation. This is largely due to a higher frequency of the more fragile bones of young individuals.

**Table 2:** Alterations recorded (%) on the *Palangana*, *Bear*, *33B*, the two Tulugak sites (*TL2* & *TL3*), *Kakinya*, *Rulland*, and *Tulukana* faunal remains.

	Palangana	Bear	33B	TL2	TL3	Kakinya	Rulland	Tulukana
not observable	0.7	0.2	1.4	9.8	3.1	4.4	2	11.2
longitudinal cracks	2.2	0.5	9.3	36.1	7.8	4.8	23.9	42
desquamation	5	4.7	13.6	43.7	14.7	6.6	20.8	46
dissolution	3.2	4	10	13.2	3.7	0.1	2.5	2.4
root etching	6.9	0.1	24.3	22.2	35.5	33.9	17.8	7.9
sheeting	1.8	0	0.7	0	0	0.6	0	30.5
carnivore damage	1.1	10	12.8	20.1	7.4	4.5	5.1	1.8
*NISP*	*277*	*1639*	*140*	*144*	*893*	*2004*	*197*	*2547*

All identified species are included.

### 3.2 Cut mark analysis

**Fig 5:**
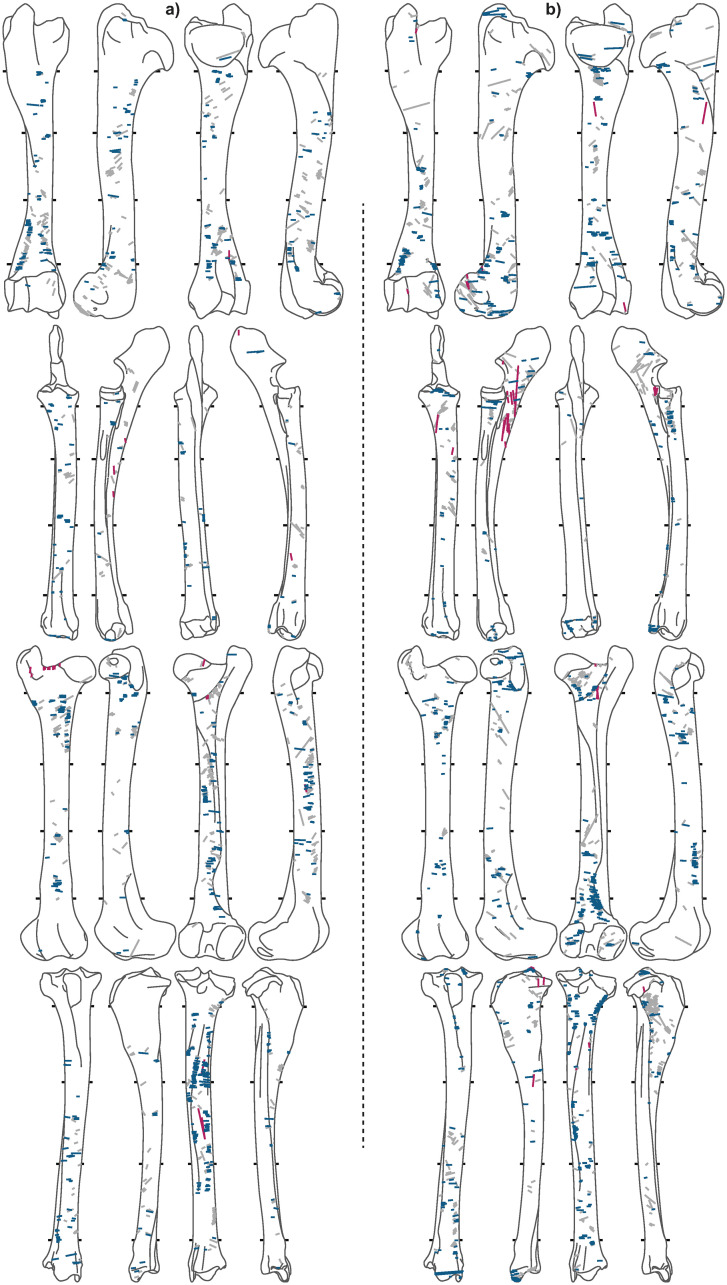
Cut mark distributions for a) *Palangana* and b) *Bear*. Red = longitudinal, blue = transverse, grey = oblique.

**Fig 6:**
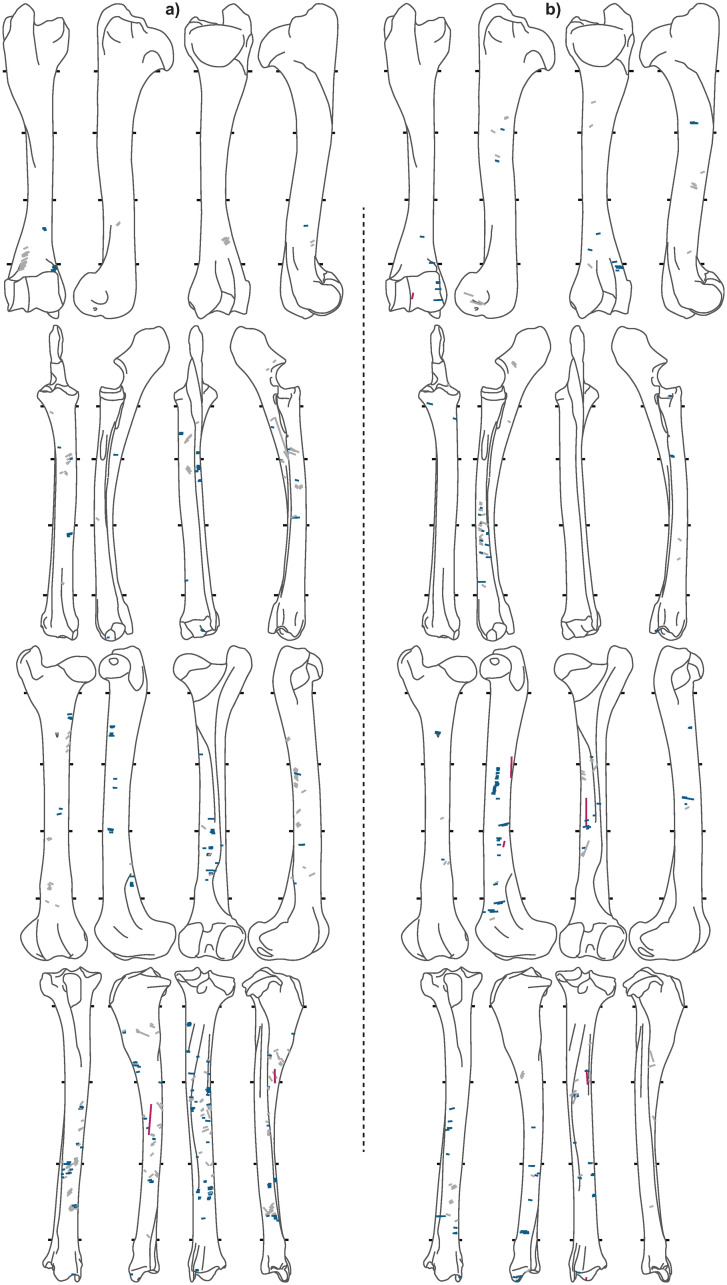
Cut mark distributions for a) *33B* and b) *TL2*. Red = longitudinal, blue = transverse, grey = oblique.

**Fig 7:**
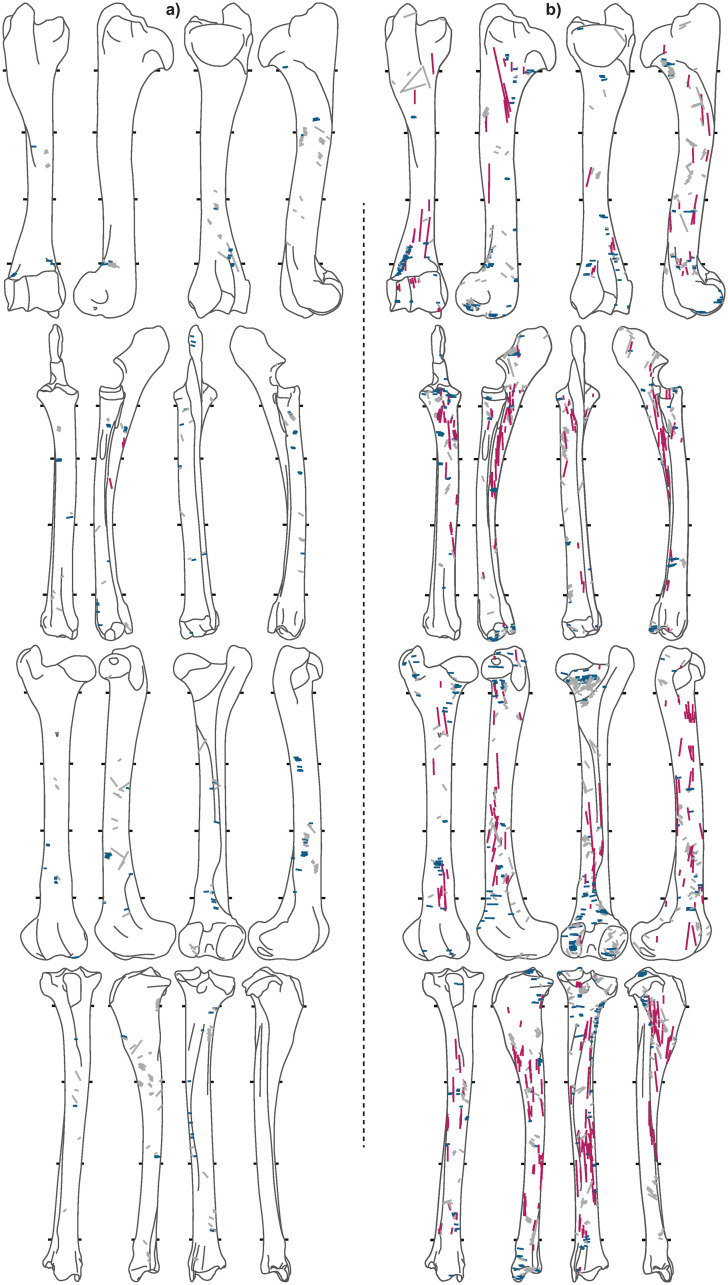
Cut mark distributions for a) *TL3* and b) *Kakinya*. Red = longitudinal, blue = transverse, grey = oblique.

**Fig 8:**
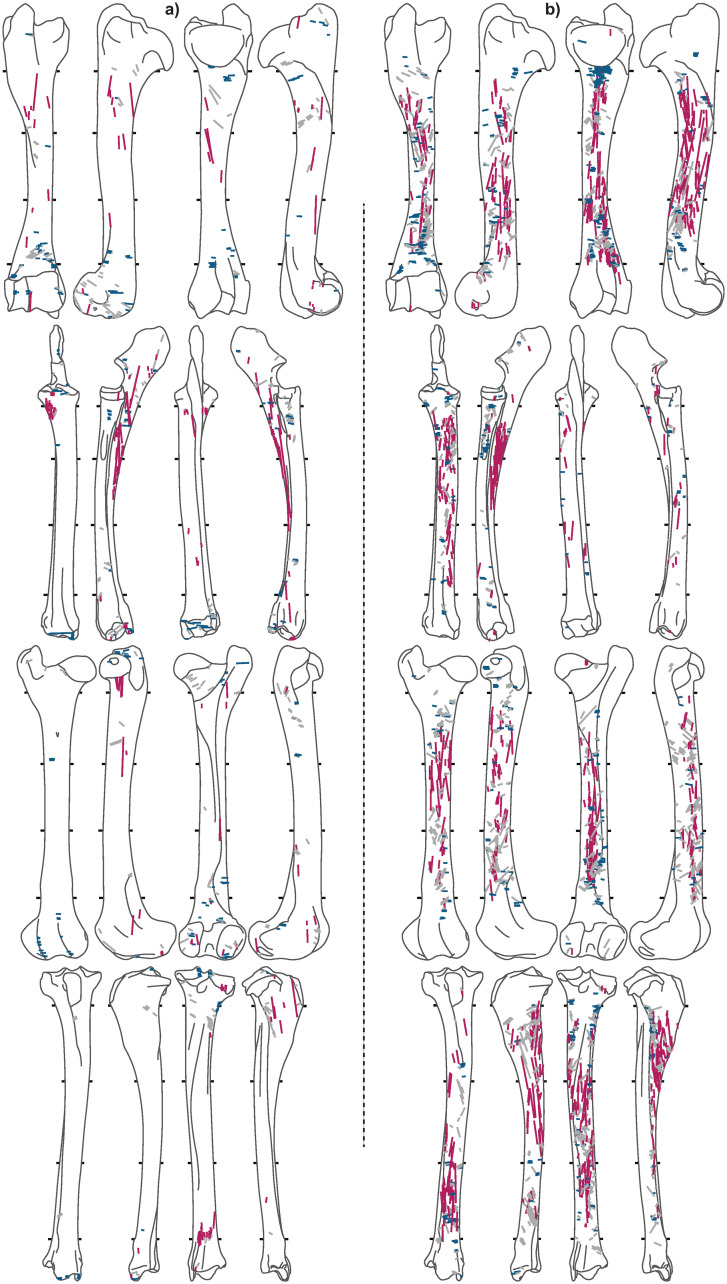
Cut mark distribution for a) *Rulland* and b) *Tulukana*. Red = longitudinal, blue = transverse, grey = oblique.

Of the 7,586 caribou bones analyzed, 2,944 are from meaty long-bones (Table 3). A total of 10,011 cut marks were recorded on long bones (Figs 5–8), of which the *Bear* and *Tulukana* sites alone account for almost half.

**Table 3:** Assemblage characteristics by site, showing the number of identified caribou remains (NISP tot.), the number of caribou remains with observable surfaces (NISP obs.), and the number of caribou remains bearing cut marks (NISP cut).

		Palangana	Bear	33B	TL2	TL3	Kakinya	Rulland	Tulukana
All bones	*NISP tot*.	277	1637	136	140	882	1788	190	2536
	*NISP obs*.	275	1602	134	126	854	1698	183	2219
	*NISP cut*	205	554	102	77	416	681	123	1129
	*cuts/obs*.	**74.5%**	**34.6%**	**76.1%**	**61.1%**	**48.7%**	**40.1%**	**67.2%**	**50.9%**
Humerus	*NISP tot*.	66	94	6	9	28	154	32	241
	*NISP obs*.	66	94	6	8	28	145	31	208
	*NISP cut*	48	64	3	6	16	52	22	141
	*cuts/obs*.	**72.7%**	**68.1%**	**50.0%**	**75.0%**	**57.1%**	**35.9%**	**71.0%**	**67.8%**
Radioulna	*NISP tot*.	87	89	12	10	40	236	22	347
	*NISP obs*.	85	89	12	9	39	224	22	301
	*NISP cut*	45	61	12	8	22	83	17	127
	*cuts/obs*.	**52.9%**	**68.5%**	**100.0%**	**88.9%**	**56.4%**	**37.1%**	**77.3%**	**42.2%**
Femur	*NISP tot*.	68	122	9	16	28	108	33	279
	*NISP obs*.	68	122	9	15	26	105	33	243
	*NISP cut*	54	80	8	8	18	73	25	140
	*cuts/obs*.	**79.4%**	**65.6%**	**88.9%**	**53.3%**	**69.2%**	**69.5%**	**75.8%**	**57.6%**
Tibia	*NISP tot*.	56	144	36	21	35	172	16	328
	*NISP obs*.	56	144	36	20	33	164	16	282
	*NISP cut*	37	98	31	16	15	77	13	161
	*cuts/obs*.	**66.1%**	**68.1%**	**86.1%**	**80.0%**	**45.5%**	**47.0%**	**81.3%**	**57.1%**

The ‘all bones’ rows correspond to all caribou remains (e.g. phalanges, femur, carpals, ribs).

Despite important differences in the size of faunal assemblages between sites, the number of cut-marked bones is consistently high, reaching more than 70% for certain skeletal elements. Cut mark frequencies do, however, vary according to skeletal element and site. No clear pattern reflecting different meat processing techniques is apparent in the ratio of cut marks to bones per site (Table 4). The average number of cut marks per fragment is highest at Rulland and 33B sites. These two sites were occupied at distinct seasons for different purposes.

**Table 4:** Number of cut marks recorded versus number of humerus, radioulna, femur and tibia.

	Palangana	Bear	33B	TL2	TL3	Kakinya	Rulland	Tulukana
N cuts QGIS	1415	2033	436	227	380	1889	817	2814
N frags QGIS	277	449	63	56	131	670	103	1195
*Ratio*	*5*.*1*	*4*.*5*	*6*.*9*	*4*.*1*	*2*.*9*	*2*.*8*	*7*.*9*	*2*.*4*

**Fig 9:**
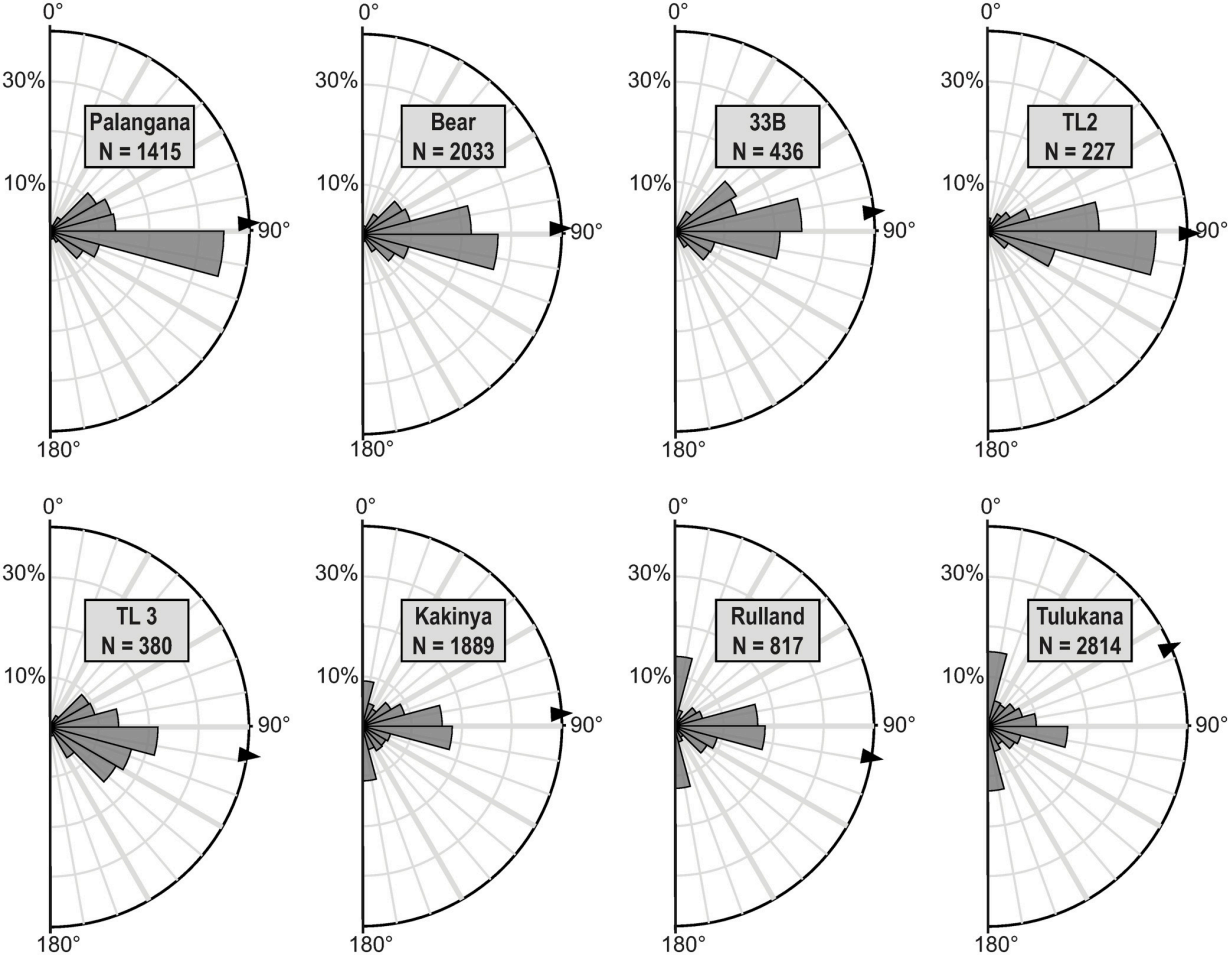
Rose diagrams for cut marks orientations for *Palangana*, *Bear*, *33B*, the two Tulugak sites (*TL2* & *TL3*), *Kakinya*, *Rulland* and *Tulukana*. The arrow indicates the mean angle.

A comparison of cut mark frequencies and orientations (Table 5) reveals oblique cut marks to be most common, except at *Bear* and *TL2* where transverse marks dominate (raw data for cut mark frequencies by long bone are available in the S1 Appendix). The prevalence of oblique cut marks is partly due by the fact that this category subsumes 8 cut mark orientation classes whereas longitudinal and transverse classes each combine only two 15°classes (see rose diagrams in Fig 9 for more detailed information concerning orientation distributions).

**Table 5:** Distribution of cut marks orientations by site and portion.

	Palangana	Bear	33B	TL2	TL3	Kakinya	Rulland	Tulukana
Tot. Portion 1	125	514	2	9	17	437	197	212
*Longitudinal*	11.2%	4.9%	-	-	-	11.9%	21.3%	14.6%
*Oblique*	44.8%	48.8%	100.0%	77.8%	64.7%	47.4%	43.7%	38.7%
*Transverse*	44.0%	46.3%	-	22.2%	35.3%	40.7%	35.0%	46.7%
Tot. Portion 2	413	497	94	33	121	398	210	942
*Longitudinal*	1.2%	2.8%	1.1%	3.0%	1.7%	32.2%	38.1%	29.4%
*Oblique*	48.2%	56.3%	51.1%	54.5%	66.1%	48.2%	41.0%	44.9%
*Transverse*	50.6%	40.8%	47.9%	42.4%	32.2%	19.6%	21.0%	25.7%
Tot. Portion 3	302	183	165	83	85	241	30	600
*Longitudinal*	1.7%	-	0.6%	2.4%	1.2%	33.6%	70.0%	38.7%
*Oblique*	47.0%	60.1%	61.2%	36.1%	71.8%	41.5%	26.7%	44.3%
*Transverse*	51.3%	39.9%	38.2%	61.4%	27.1%	24.9%	3.3%	17.0%
Tot. Portion 4	451	462	158	74	121	431	136	951
*Longitudinal*	0.4%	0.2%	-	1.4%	-	19.7%	23.5%	24.1%
*Oblique*	55.4%	41.3%	48.1%	43.2%	57.9%	46.6%	34.6%	50.7%
*Transverse*	44.1%	58.4%	51.9%	55.4%	42.1%	33.6%	41.9%	25.2%
Tot. Portion 5	124	377	17	28	36	382	244	109
*Longitudinal*	-	1.6%	-	7.1%	-	8.1%	16.4%	12.8%
*Oblique*	49.2%	40.8%	23.5%	21.4%	52.8%	44.0%	36.5%	46.8%
*Transverse*	50.8%	57.6%	76.5%	71.4%	47.2%	47.9%	47.1%	40.4%

When oblique cut marks are excluded, cut mark distributions clearly highlight different patterns between sites; longitudinal cut marks are rare (< 2.2%) while transverse cut marks are abundant in all winter and summer campsites (Figs 5, 6 & 7a), where stored frozen and freshly slaughtered meat was, respectively, consumed. Conversely, *Kakinya* (Fig 7b), *Rulland* (Fig 8a) and *Tulukana* (Fig 8b), all occupied during the fall, have higher frequencies of longitudinal cut marks (> 27%) and lower frequencies of transverse cut marks (Fig 9).

Data from spongy portions 1 and 5 should be considered with caution, as disarticulation is most likely to leave cut marks, including longitudinal marks, on these portions rather than on shafts [54, 61]. Taphonomic processes, cooking, and the use of these spongy portions as fuel can also lead to their destruction [e.g. 78–81]. In the present case, while articular extremities are rare compared to shaft portions at most sites, they are overrepresented at *Bear*, *Rulland*, and *Kakinya* (see S2 Appendix), which Binford’s informants mention as being due to their storage in the event of a shortage in fat. When portions 1 and 5 (Table 6) are excluded, the overall pattern remains the same: oblique cut marks are most frequent, and sites occupied in the autumn have the highest number of longitudinal cut marks. However, when these portions are excluded 1) the overall number of transverse cut marks decreases, except for a slight increase at *Palangana*), 2) longitudinal cut marks at *Palangana*, *Bear*, and *TL2* decrease, and 3) longitudinal cut marks increase, especially at the autumn campsites of *Kakinya*, *Rulland*, and *Tulukana*.

**Table 6:** Percentage of cut mark orientation for shaft portions.

		Palangana	Bear	33B	TL2	TL3	Kakinya	Rulland	Tulukana
All portions	Longitudinal	1.84	2.26	0.46	2.64	0.79	19.96	26.32	27.83
	Oblique	50.04	48.50	52.98	40.97	63.42	45.95	38.68	46.34
	Transverse	48.13	49.24	46.56	56.39	35.79	34.09	35.01	25.84
Portions 1 & 5 excluded	Longitudinal	1.03	1.31	0.48	2.11	0.92	27.48	35.37	29.60
	Oblique	50.69	50.88	53.96	42.11	64.53	46.07	37.50	46.97
	Transverse	48.28	47.81	45.56	55.79	34.56	26.45	27.13	23.43

The percentages of fragments from portions 2 to 4 with at least one longitudinal cut mark evince a comparable pattern (Table 7): autumn sites (*Kakinya*, *Rulland* and *Tulukana*) have overall and per-skeletal element %cutL that systematically exceeds 45%, while the %cutL is always below 25% and most often under 10% in the other sites.

**Table 7:** %cutL for fragments from portions 2 to 4 with at least one longitudinal cut mark.

	**Palangana**			**Bear**			**33B**			**TL2**		
	N. frag. cutL	N. fragC	**%cutL**	N. frag. cutL	N. fragC	**%cutL**	N. frag. cutL	N. fragC	**%cutL**	N. frag. cutL	N. fragC	**%cutL**
Humerus	1	48	**2.08**	7	64	**10.94**	0	3	**0**	1	6	**0.17**
Radioulna	4	45	**8.89**	11	61	**18.03**	0	12	**0**	0	8	**0**
Femur	4	54	**7.41**	5	80	**6.25**	0	8	**0**	2	8	**25.00**
Tibia	2	37	**5.41**	7	98	**7.14**	2	31	**6.45**	2	16	**12.50**
*All*	*11*	*184*	***5*.*98***	*30*	*303*	***9*.*90***	*2*	*54*	***3*.*70***	*5*	*38*	***13*.*16***
	**TL3**			**Kakinya**			**Rulland**			**Tulukana**		
	N. frag. cutL	N. fragC	**%cutL**	N. frag. cutL	N. fragC	**%cutL**	N. frag. cutL	N. fragC	**%cutL**	N. frag. cutL	N. fragC	**%cutL**
Humerus	0	16	**0**	25	52	**48.08**	15	22	**68.18**	70	141	**49.65**
Radioulna	2	22	**9.09**	39	83	**46.99**	11	17	**64.71**	63	127	**49.61**
Femur	0	18	**0**	36	73	**49.32**	15	25	**60.00**	58	140	**41.43**
Tibia	0	15	**0**	38	77	**49.35**	9	13	**69.23**	87	161	**54.04**
*All*	*2*	*71*	***2*.*82***	*138*	*285*	***48*.*40***	*50*	*77*	***64*.*94***	*278*	*569*	***48*.*90***

Column legend: N. frag cutL = Number of fragments with at least one longitudinal cut mark, N. fragC = Number of fragments with cut marks.

The length classes demonstrates ‘intermediate’ cut marks to be most frequent, comprising more than 94% of all traces in each assemblage (Table 8). Short cut marks are uncommon and ‘long’ examples are even more rare, however the latter class includes less size classes than the two others. Excluding portions 1 and 5 (Table 8) reduces the number of short cut marks in 5 of 8 cases, and increases the percentage of long cut marks (6 out of 8 cases) as well as the proportion of ‘long’ cut marks in 5 cases. Independent of whether portions 1 and 5 are considered; these ‘long cut marks’ are more common in the *Kakinya*, *Rulland* and *Tulukana* assemblages.

**Table 8:** Distribution of cut marks by length class for *Palangana*, *Bear*, *33B*, the two Tulugak sites (*TL2* & *TL3*), *Kakinya*, *Rulland*, and *Tulukana*.

		Palangana	Bear	33B	TL2	TL3	Kakinya	Rulland	Tulukana
All portions	short	1.77	4.57	4.36	1.76	1.32	2.91	3.67	0.75
	intermediate	97.95	94.84	95.18	97.80	98.42	95.08	94.25	95.81
	long	0.28	0.59	0.46	0.44	0.26	2.01	2.08	3.45
Portions 1 & 5 excluded	short	1.72	4.64	4.34	2.11	1.53	2.06	1.60	0.72
	intermediate	98.03	94.83	95.23	97.37	98.17	94.49	94.95	95.39
	long	0.26	0.53	0.43	0.53	0.31	3.46	3.46	3.89

## 4. Discussion

The study of the Nunamiut faunal material collected by L. Binford provides several lines of evidence for better identifying and interpreting patterns of food storage and consumption.

### 4.1 Skilled butchers equally produce high numbers of cut marks

One of the first things worth mentioning is the high number of cut marks recorded for all campsites. It is widely agreed that cut mark frequency varies as a function of the butcher’s skill: the more experienced a butcher is, the less cut marks they leave on bones [50, 82]. This correlation has primarily been suggested for butchers using metal knives due to the greater care paid to the blade’s edge compared to an easily replaceable un-retouched stone tool [83]. In this study, at least 33% of bones with observable cortical surfaces from all 8 campsites bear at least one cut mark. It is worth noting that metal knives were highly valued and looked after by northern Alaskan Nunamiut groups, as sourcing manufactured materials was difficult until recently. This unbiased ethnographic collection produced by experienced individuals using traditional techniques as part of their daily routine rather than experimentally derived data does not support the hypothesis that skilled butchers produce less cut marks than novices or less experienced individuals. Despite the use of metal knives, cut marks are very frequent, found on *at least* a third of bone remains with observable surfaces.

### 4.2 Distinct cut marks patterns according to carcass preparation

Campsites occupied during different seasons and dedicated to specific food-related activities allow potential correlations between cut mark patterns and carcass processing to be explored. Our data show that autumn campsites where carcasses were processed for the production of dried-meat have significant numbers (more than 20%) of longitudinal cut marks. Conversely, this pattern of cut marks is not observed (less than 3% of the cut marks are longitudinally oriented) on bones recovered from sites where carcasses were processed using techniques commonly linked with the immediate consumption of meat on summer campsites. This pattern is equally the case in sites where the prepared portions of carcasses had been preserved in ice-cellars. The connection between a significant number of longitudinal cut marks and meat drying is further strengthened by the fact that our data from the butchery of caribou in northern Alaska are consistent with what was documented for antelope remains from South Africa [62]. The preparation of meat for drying generates longitudinal cut marks in large proportions, which is further reinforced by the fact that, in the present study, this pattern remains consistent for sites occupied in the 1950s as well as those from the late 19^th^ century. To a lesser extent, the same pattern emerges when cut mark length is considered. ‘Long’ cut marks are more frequent in sites where meat was dried.

High frequencies of longitudinal and long cut marks is therefore only documented for meat drying and not for other butchery activities. This pattern is stable over time and between species and consistent in very different parts of the world amongst groups with substantially different cultural traditions and who occupied regions with drastically contrasting environmental, climatic, and topographical conditions.

It should be pointed out that these results may only apply to artiodactyls. The particular morphologies of bones from other species, for example, perissodactyls with their 3^rd^ trochanter, may require different butchery techniques and hence produce different cut mark orientations and frequencies. Moreover, cut marks on articular extremities should be treated with caution when exploring defleshing techniques, given certain dismemberment techniques can equally produce large numbers of longitudinal marks.

### 4.3 Same butcher: Distinct activities, contrasting patterns

A comparison of data from the *Kakinya* and *Bear* sites demonstrates cut mark patterns to reflect the type of preparation carried out and not different cultural traditions or know-how. Butchery activities were conducted by the same person (Unalîna, Elidja Kakinya’s wife) at both sites; however, at *Kakinya* meat was prepared for drying while at the winter *Bear* site meat previously stored in ice-cellars was processed and consumed. Cut mark patterns identified at these two sites are radically different (see Figs 5 & 7). At *Kakinya*, 20% of the cut marks are longitudinally oriented (27.5% if only shaft fragments are considered) and almost half of the cut-marked bones bear at least one longitudinal mark. In contrast, at the winter *Bear* campsite, where meat from ice-cellars was processed, less than 2.5% (and 1.3% when only shaft fragments are considered) of the cut marks are longitudinal and no more than 10% of the bones have longitudinal marks.

### 4.4 The %cutL: A quicker alternative to geo-referencing

Mapping cut marks is long and tedious, and it is not surprising that the method is seldom incorporated in zooarchaeology. Nevertheless, as demonstrated here, a detailed analysis of cut marks produced during defleshing provides valuable insights for documenting meat processing techniques used by past human societies. The easily calculated %cutL presented here is a reliable, time-saving alternative to cut mark digitalization and mapping, and can be easily integrated in zooarchaeological analysis. In fact, these data may already be in many zooarchaeologist’s databases. This index has recently been integrated in the TIPZOO graphical interface and can be calculated automatically [84]. This study has shown that at all three sites where meat was dried, the %cutL exceeds 45% and rarely reaches 10% with other meat processing types.

## 5. Conclusion

Food is a fundamental, everyday element of all societies, and exploring consumption patterns and eating habits of past societies sheds important light on a wide range of issues surrounding both human evolution as well as culture, social relations, and group organization. Food practices are governed by a whole series of rites and taboos that constitute the specific codes that define each society. Our evaluation of cut mark patterns from a large ethnographic rather than experimentally produced faunal assemblage offers important perspectives for exploring past hunter-gatherer societies, their eating habits, and, by extension, their mobility systems as well as aspects of social relations and organization. Whether it concerns the production of dried-meat, the storage of frozen food or the defleshing of a carcass for immediate consumption, identifying modes of meat preparation provides a window onto the socio-economic organization of past societies, particularly nomadic ones.

## Supporting information

S1 Appendix QGIS files, with long bone fragments and raw data on cut marks. (XLSX)

S2 Appendix Humerus, radioulna, femur and tibia bone remains recorded in QGIS. (PDF)
