# Supplementary material for: Exploring meat processing in the past: Insights from the Nunamiut people
Source: PLoS One. 2021 Jan 13;16(1):e0245213. doi: 10.1371/journal.pone.0245213 (PMC7806178; doi:10.1371/journal.pone.0245213)
Supplement: S2 Appendix — (PDF) [file pone.0245213.s002.pdf]

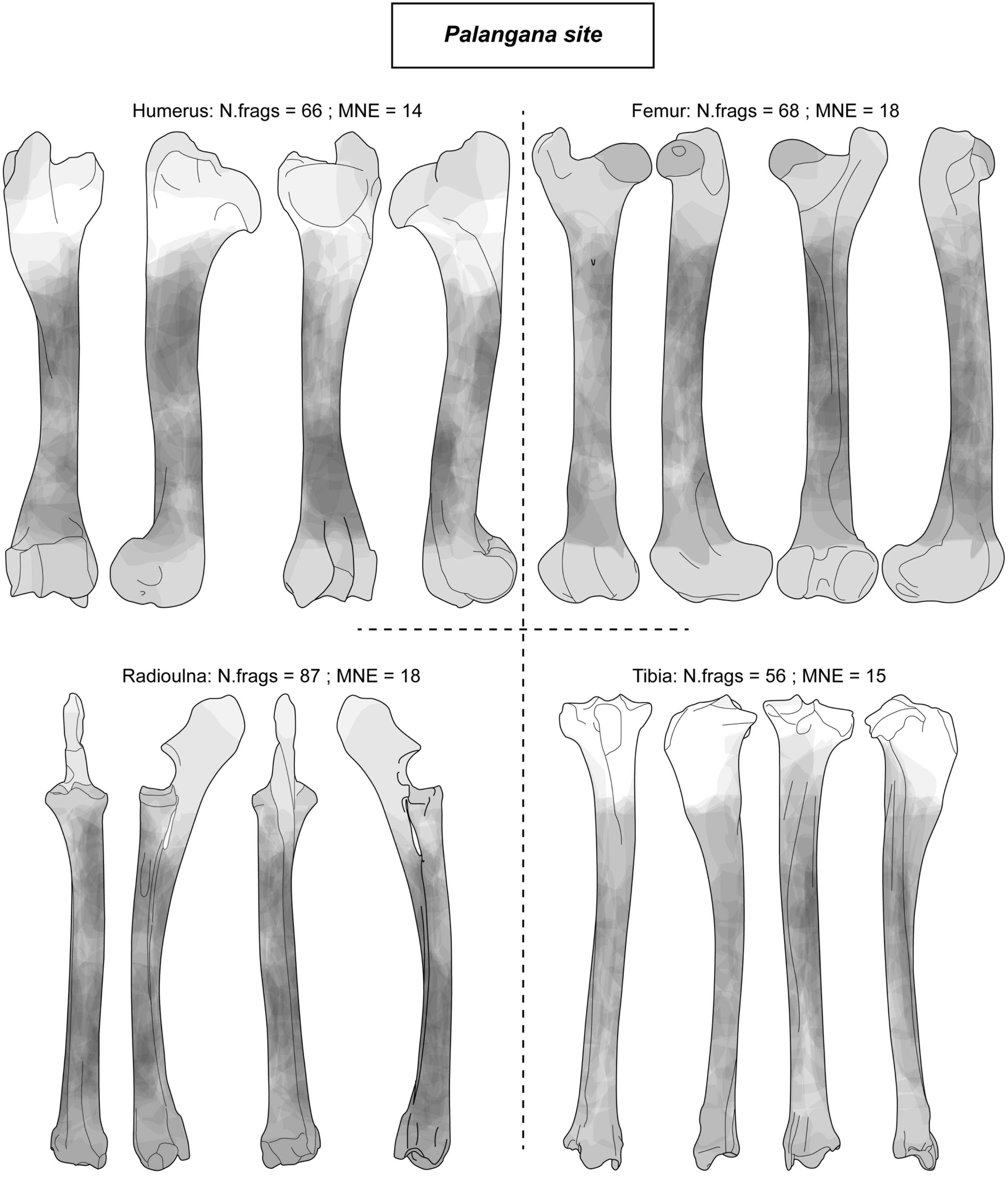
Palangana site
Humerus: N.frags = 66 ; MNE = 14
Femur: N.frags = 68 ; MNE = 18
Radioulna: N.frags = 87 ; MNE = 18
Tibia: N.frags = 56 ; MNE = 15

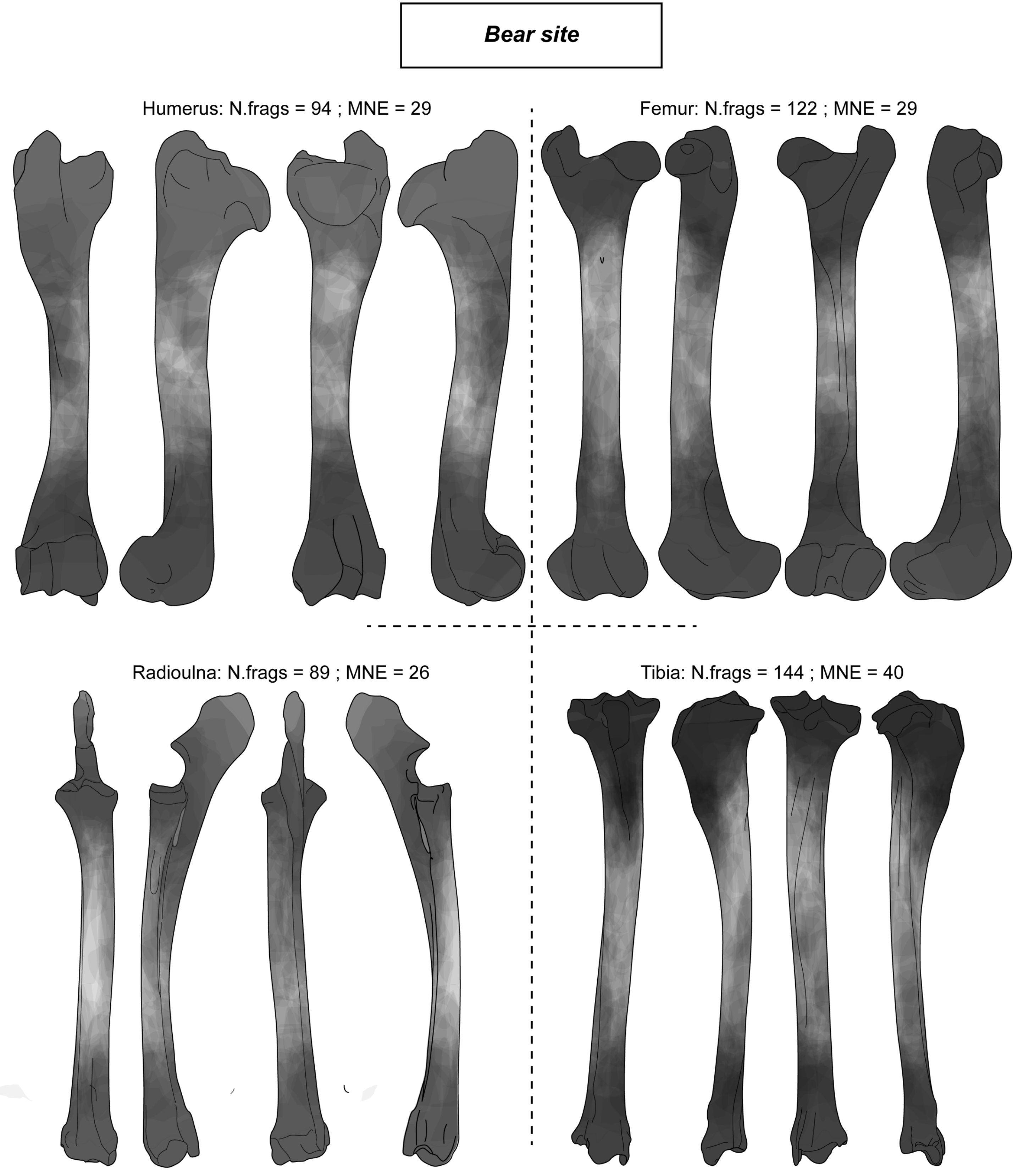
Bear site
Humerus: N.frags = 94 ; MNE = 29
Femur: N.frags = 122 ; MNE = 29
Radioulna: N.frags = 89 ; MNE = 26
Tibia: N.frags = 144 ; MNE = 40

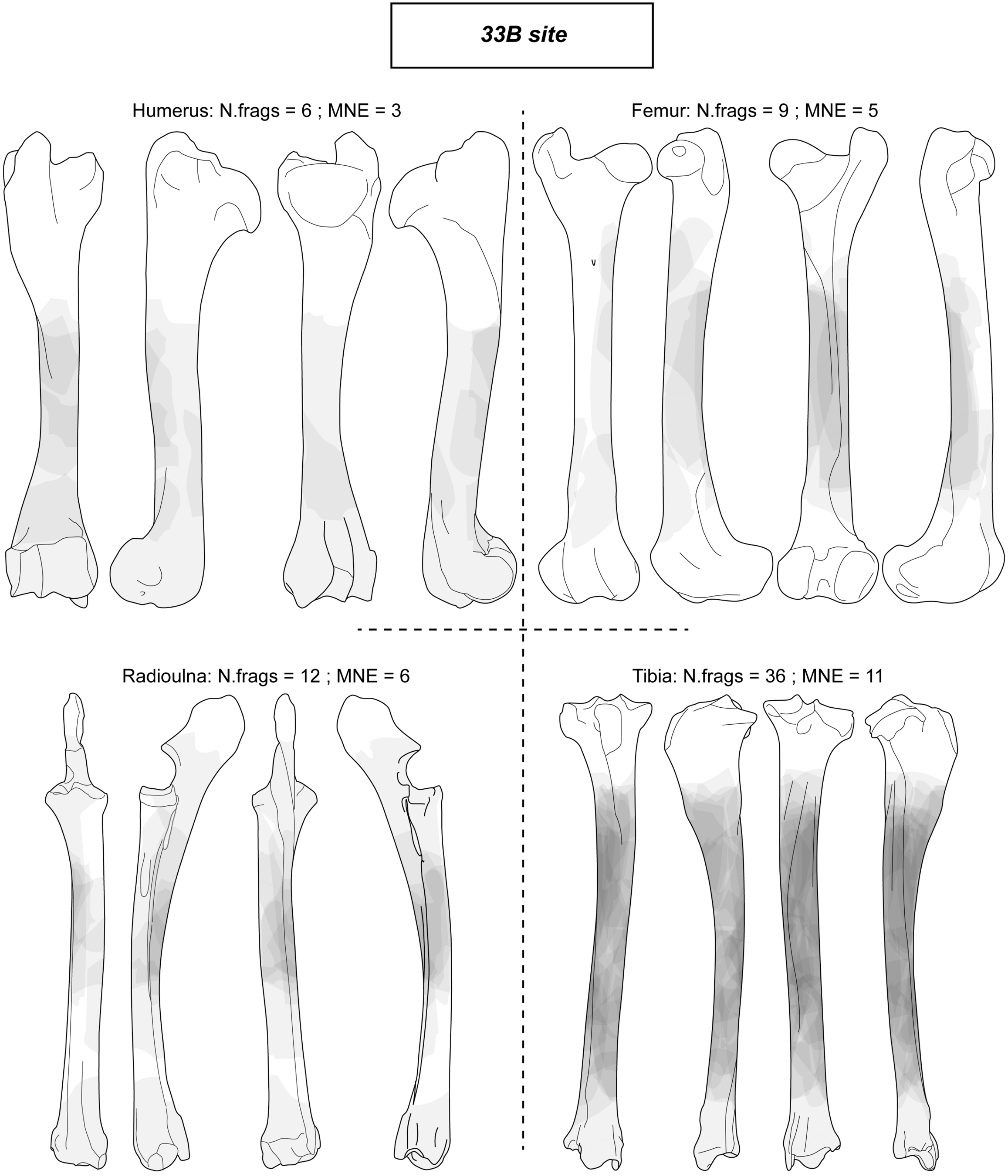
33B site
Humerus: N.frags = 6 ; MNE = 3
Femur: N.frags = 9 ; MNE = 5
Radioulna: N.frags = 12 ; MNE = 6
Tibia: N.frags = 36 ; MNE = 11

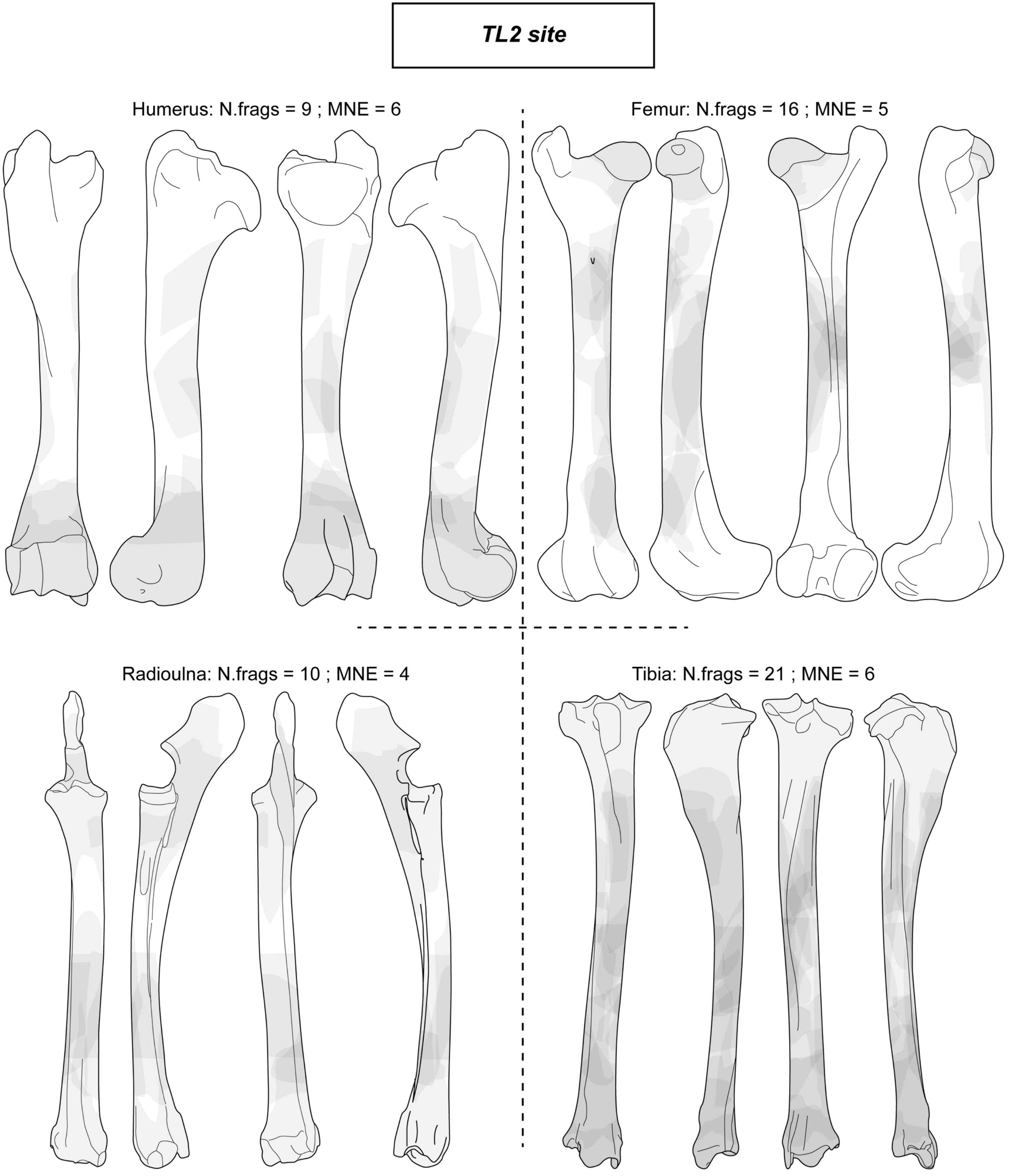
TL2 site
Humerus: N.frags = 9 ; MNE = 6
Femur: N.frags = 16 ; MNE = 5
Radioulna: N.frags = 10 ; MNE = 4
Tibia: N.frags = 21 ; MNE = 6

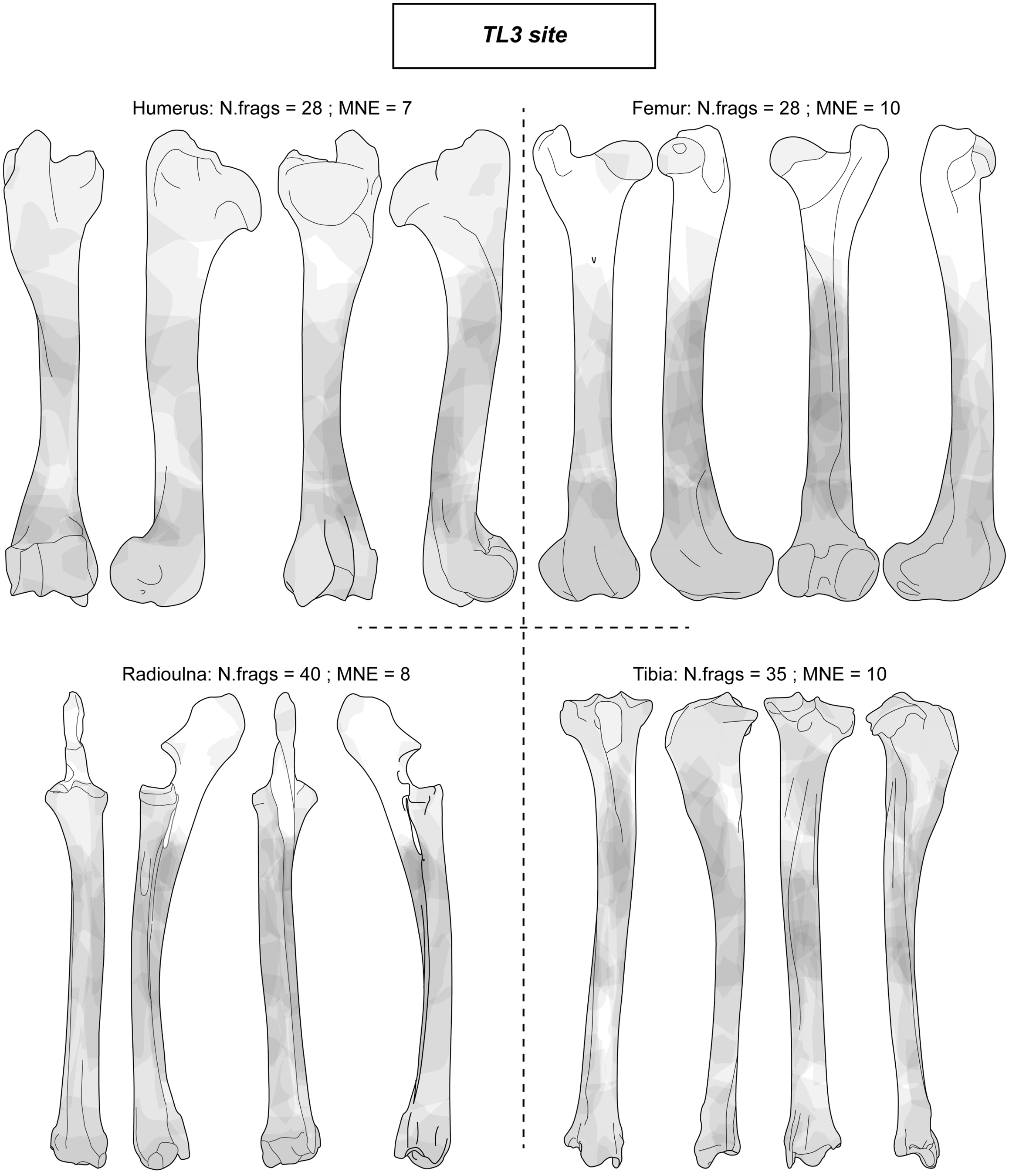
TL3 site
Humerus: N.frags = 28 ; MNE = 7
Femur: N.frags = 28 ; MNE = 10
Radioulna: N.frags = 40 ; MNE = 8
Tibia: N.frags = 35 ; MNE = 10

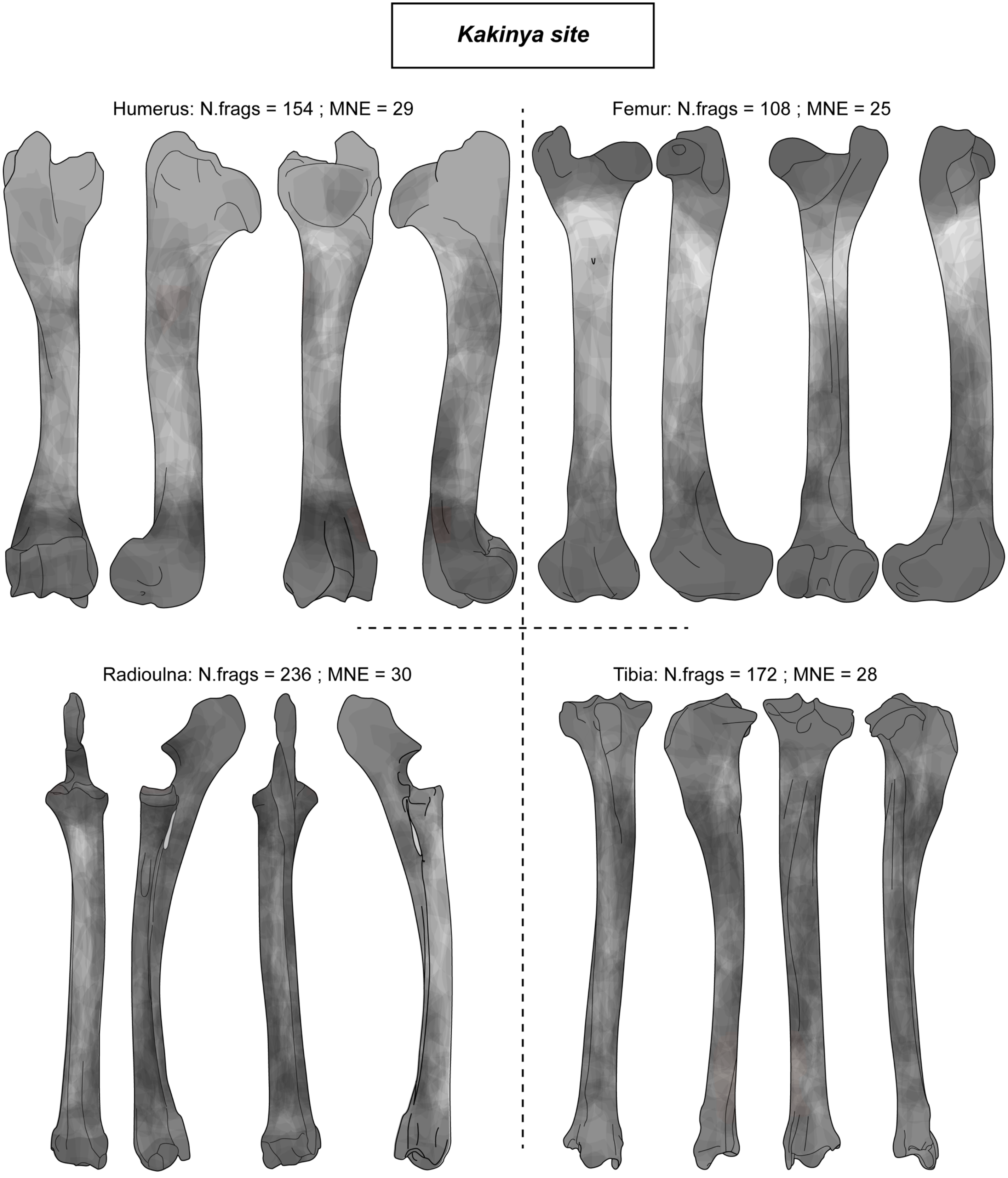
Kakinya site
Humerus: N.frags = 154 ; MNE = 29
Femur: N.frags = 108 ; MNE = 25
Radioulna: N.frags = 236 ; MNE = 30
Tibia: N.frags = 172 ; MNE = 28

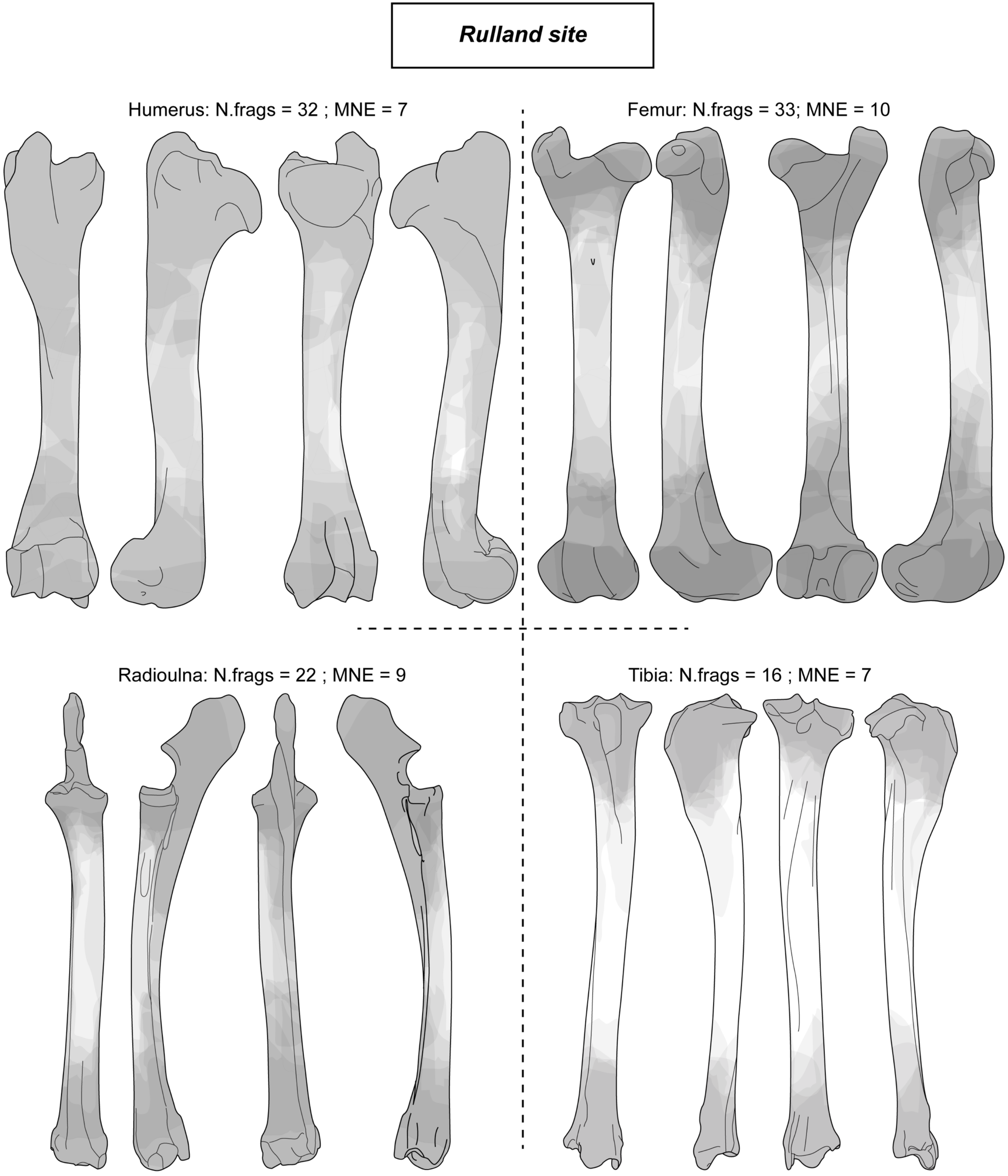
Rulland site
Humerus: N.frags = 32 ; MNE = 7
Femur: N.frags = 33; MNE = 10
Radioulna: N.frags = 22 ; MNE = 9
Tibia: N.frags = 16 ; MNE = 7

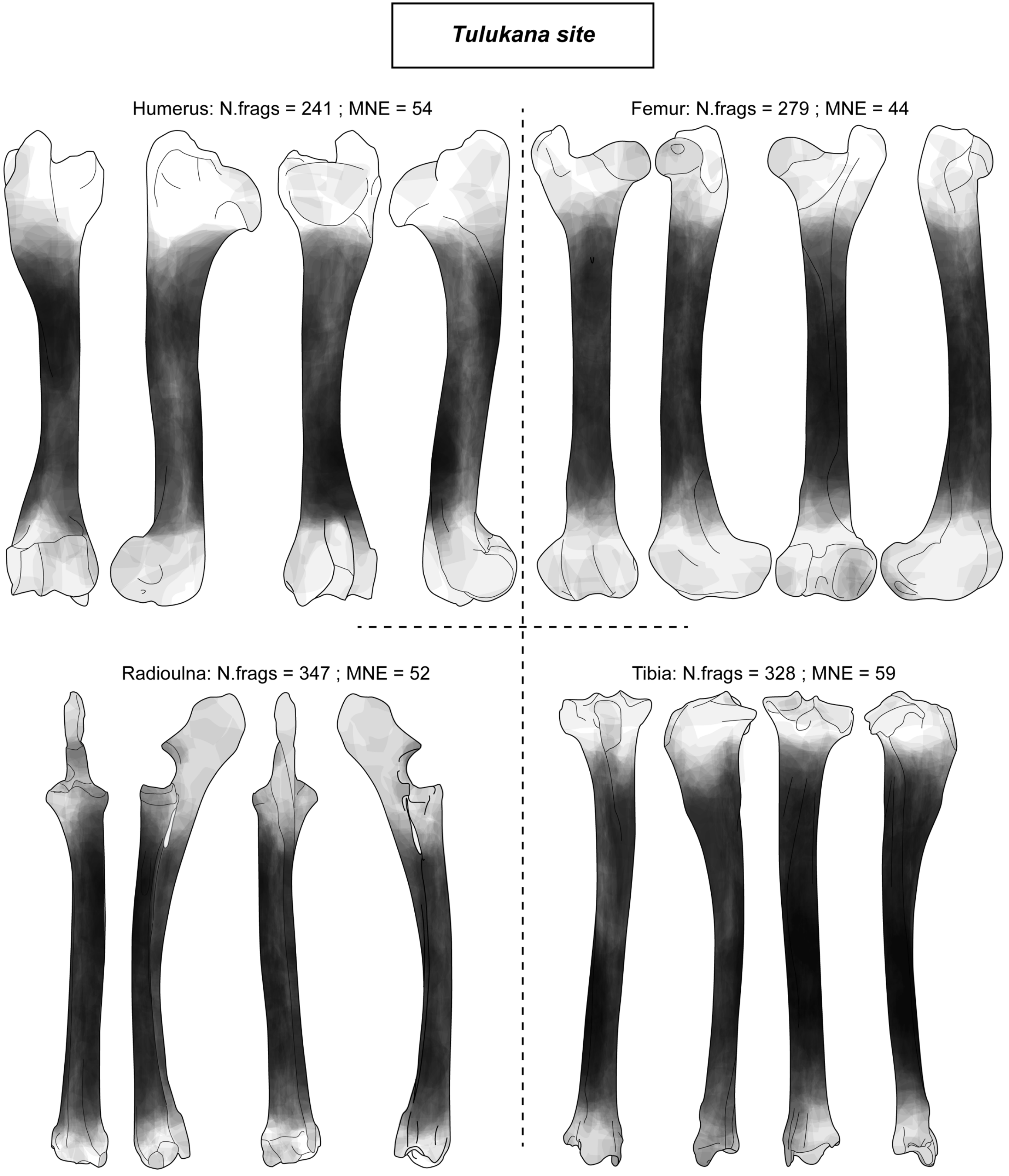
Tulukana site
Humerus: N.frags = 241 ; MNE = 54
Femur: N.frags = 279 ; MNE = 44
Radioulna: N.frags = 347 ; MNE = 52
Tibia: N.frags = 328 ; MNE = 59
